# Rilpivirine Activates STAT1 in Non-Parenchymal Cells to Regulate Liver Injury in People Living with HIV and MASLD

**DOI:** 10.3390/biomedicines12071454

**Published:** 2024-06-29

**Authors:** Ángela B. Moragrega, Carmen Busca, Nadezda Apostolova, Antonio Olveira, Luz Martín-Carbonero, Eulalia Valencia, Victoria Moreno, José I. Bernardino, Marta Abadía, Juan González-García, Juan V. Esplugues, María L. Montes, Ana Blas-García

**Affiliations:** 1 Departamento de Farmacología, Universitat de València, Av. Blasco Ibáñez, 15, 46010 Valencia, Spain; angela.moragrega@uv.es (Á.B.M.); nadezda.apostolova@uv.es (N.A.); juan.v.esplugues@uv.es (J.V.E.); 2 FISABIO (Fundación para el Fomento de la Investigación Sanitaria y Biomédica de la Comunidad Valenciana), Av. de Catalunya, 21, 46020 Valencia, Spain; 3 Unidad VIH, Servicio de Medicina Interna, Hospital Universitario La Paz, Institute for Health Research (IdiPAZ), 28046 Madrid, Spain; carmen.busca@gmail.com (C.B.); lmcarbonero@gmail.com (L.M.-C.); eulalia.valencia@salud.madrid.org (E.V.); vmoreno.hciii@salud.madrid.org (V.M.); naberse@gmail.com (J.I.B.); juangonzalezgar@gmail.com (J.G.-G.); mmontesr2001@yahoo.es (M.L.M.); 4 CIBERINFEC (Centro de Investigación Biomédica en Red de Enfermedades Infecciosas), Instituto de Salud Carlos III, 28046 Madrid, Spain; 5 CIBEREHD (Centro de Investigación Biomédica en Red de Enfermedades Hepáticas y Digestivas), Instituto de Salud Carlos III, 46010 Valencia, Spain; 6 Servicio de Aparato Digestivo, Hospital Universitario La Paz, 28046 Madrid, Spain; aolveiram@gmail.com (A.O.); mabadiab@gmail.com (M.A.); 7 Departamento de Fisiología, Universitat de València, Av. Blasco Ibáñez, 15, 46010 Valencia, Spain

**Keywords:** fibrosis, MASH, hepatic stellate cells, antiretroviral therapy

## Abstract

Liver fibrosis is a key determinant of the progression of metabolic dysfunction-associated steatotic liver disease (MASLD). Its increasing prevalence and a lack of effective treatments make it a major health problem worldwide, particularly in people living with HIV, among whom the prevalence of advanced fibrosis is higher. We have published preclinical data showing that Rilpivirine (RPV), a widely used anti-HIV drug, selectively triggers hepatic stellate cell (HSC) inactivation and apoptosis through signal transducer and activator of transcription (STAT)1-mediated pathways, effects that clearly attenuate liver fibrosis and promote regeneration. We performed a retrospective, cross-sectional study of RPV-induced effects on steatosis, inflammation, and fibrosis in liver biopsies from well-controlled HIV-infected subjects diagnosed with MASLD. Patients on RPV exhibited similar levels of HIV-related parameters to those not receiving this drug, while showing a tendency toward improved liver function and lipid profile, as well as an enhanced activation of STAT1 in hepatic non-parenchymal cells in those with identified liver injury. This protective effect, promoting STAT1-dependent HSC inactivation, was observed at different stages of MASLD. Our results suggest that RPV-based therapy is especially indicated in HIV-infected patients with MASLD-derived liver injury and highlight the potential of RPV as a new therapeutic strategy for liver diseases.

## 1. Introduction

Liver fibrosis is a wound-healing response characterized by excessive extracellular matrix deposition during chronic liver injury and is mediated mainly by activation of hepatic stellate cells (HSCs). As a key determinant of metabolic dysfunction-associated steatotic liver disease (MASLD) progression and clinical prognosis, it constitutes a major health problem due to its rapidly increasing prevalence and a lack of specific, effective treatments [1]. Moreover, MASLD is a major cause of chronic liver disease in people living with HIV (PLWH), among whom there is a 10–15% prevalence of advanced liver fibrosis [2]. Recently, we have published the results of experiments in vitro and in different murine models of chronic liver disease showing that the non-nucleoside reverse transcriptase inhibitor (NNRTI) Rilpivirine (RPV), an antiretroviral drug widely used to treat HIV infection, selectively triggers HSC inactivation and apoptosis through signal transducer and activator of transcription (STAT)1-mediated pathways, effects that clearly attenuate liver fibrosis and promote regeneration [3]. These results were in line with previously reported evidence that STAT1 activation in HSCs promotes different signaling pathways involved in the attenuation of liver injury in animal models [4]. Furthermore, RPV-treated patients display a lower risk of dyslipidemia and better liver function than patients receiving RPV-free regimens [3,5]. Lipidomic studies also suggest that RPV increases plasma levels of metabolites with anti-inflammatory properties and decreases those of lipotoxic lipids in patients who switch to RPV from therapies containing efavirenz, a first generation NNRTI [6]. In addition, evidence of anti-inflammatory and immunomodulating actions of RPV in the context of chronic liver diseases has been reported using different in vivo, ex vivo, and in vitro models [7]. However, little such evidence has been obtained specifically from human liver samples. In this work, we have studied the effects of RPV on hepatic steatosis, inflammation, and fibrosis in liver biopsies from HIV-infected subjects diagnosed with MASLD.

## 2. Materials and Methods

*Patients and study design.* This retrospective, cross-sectional study was conducted in 42 well-controlled HIV-infected subjects in whom there was a clinical suspicion of MASLD and who underwent confirmatory liver biopsy (Hospital La Paz, Madrid, Spain). These patients were from a cohort of PLWH studied for increased transaminase levels for ≥6 months, who were on stable combined antiretroviral therapy (cART) and had presented <50 copies/mL of HIV-RNA for ≥1 year. The exclusion criteria were past or present chronic hepatitis B or C virus coinfection, high alcohol consumption (>30 g/d in men or ≥20 g/d in women), potential hepatotoxicity, and other liver diseases. Patients were not selected based on their sex or cART composition, and no alterations were made to their therapy after inclusion in the study. All patients underwent a screening protocol for autoimmune, genetic, and metabolic liver disease, as well as a liver ultrasound and measurement of liver stiffness and steatosis by transient elastography (TE) and controlled attenuation parameter (CAP). A liver biopsy was offered to all patients, according to EASL-EASD guidelines [8]. Approval was obtained from the ethics committee of the Hospital Universitario La Paz (Madrid, Spain; approval number PI-2248) and written informed consent was obtained from all participants.

*Variables and measurements.* Demographic, clinical, laboratory, and HIV-related data, as well as metabolic anthropometric and clinical data, were collected for each patient. We also recorded exposure to antidiabetic, antihypertensive, and lipid-lowering medications.

*Liver ultrasound and TE.* Real-time liver ultrasound was performed under fasting conditions using an Aplio 500 Platinum device (Canon Medical Systems S.A., Otawara-shi, Japan); results were expressed as the presence or absence of steatosis. TE was performed according to the manufacturer’s protocol under fasting conditions using a FibroScan device (Probe M, FS402; Echosens (Paris, France)). The CAP cut-off value for the diagnosis of steatosis was >248 dB/m [9], and liver stiffness measurements corresponding to significant (F ≥ 2) and advanced (F ≥ 3) fibrosis were ≥7.0 kPa and >9.6 kPa, respectively [10].

*Liver biopsy and histological studies.* Percutaneous liver biopsy with a 16G needle was performed according to the Menghini technique under ultrasound guidance. Liver samples were formalin-fixed and paraffin-embedded for histological analysis. We performed haematoxylin and eosin staining (steatosis was graded, based on the percentage of affected hepatocytes, as 0: 0–5%, 1: 6–33%, 2: 34–66%, and 3: 67–100%) and immunohistochemistry (IHC) with an anti-STAT1 rabbit monoclonal antibody (1:250, Cell Signaling, 14994), as previously described [3]. Steatohepatitis and fibrosis were categorized using the scoring system proposed by Kleiner et al.; liver fibrosis was classified, using the METAVIR system, as mild (F0–F2) or advanced (F3–F4) [11]. IHC images were acquired using a digital light microscope (Leica DMD 108, Leica Microsystems, Wetzlar, Germany), and quantifications were performed using Image J software V1.50i. Activation of STAT1 was assessed by the presence of its nuclear expression.

*Statistical analysis.* Variables were summarized as proportions for categorical variables and median and interquartile ranges for continuous variables. Continuous variables were analyzed using the *t* test or Mann–Whitney test, depending on the normality of the distribution (Kolmogorov–Smirnov test with a Lilliefors correction). STAT1 nuclear expression was quantified and compared—after log transformation (Ln) of values—in subjects with versus without RPV-based therapy. Differences in LnSTAT-1 were analyzed by factorial analysis of variance, considering exposure to RPV and diagnoses of steatosis, steatohepatitis, and fibrosis as inter-subject factors, and exposure time to RPV and body mass index as covariates.

## 3. Results

### 3.1. Study Population and Effects of RPV-Containing Regimens on Patient Characteristics

The study subjects who fulfilled the inclusion criteria were all male, with a median age of 49 years and a median CD4^+^ count of 802 cells/µL, and 60% of them had metabolic syndrome. All subjects presented prolonged HIV infection (>12 years) with an undetectable HIV viral load and were receiving cART; among them, 45% were receiving RPV-based therapy. Liver biopsies from our patients showed that 42.9% presented moderate or severe steatosis (>33%), 67.6% steatohepatitis, and 42.9% fibrosis (F ≥ 1); importantly, only 7.1% of subjects presented advanced liver fibrosis (F ≥ 3). Baseline characteristics of the whole population are shown in Table 1. Regarding the effects of RPV-containing regimens on patient characteristics, when compared to non RPV-exposed subjects, patients receiving this NNRTI exhibited similar levels of HIV-related parameters. There were no significant differences regarding metabolic and liver-related variables, but RPV-treated individuals showed a tendency toward lower TE and CAP measurements, and lower rates of hypercholesterolemia, hypertriglyceridemia, and treatment with lipid-lowering drugs. Importantly, a lower proportion of patients with TE > 7 kPa was detected in the group receiving RPV-containing therapy, a difference that was statistically significant (Table 1).

### 3.2. Hepatic STAT1 Activation in Patients Treated with RPV-Based Therapy

**Figure 1:**
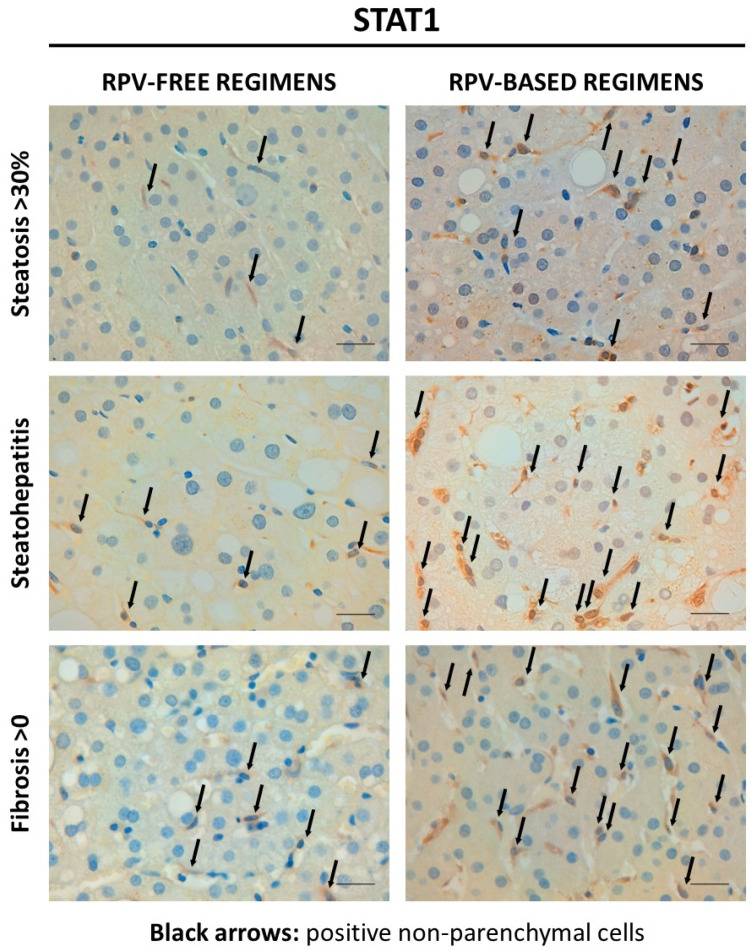
Nuclear STAT1 expression in non-parenchymal cells of liver biopsies from people living with HIV with diagnosed metabolic dysfunction-associated steatotic liver disease (different disease groups: steatosis > 30%, steatohepatitis and fibrosis > 0). Representative images of STAT1 immunohistochemistry in hepatic sections from patients with identified liver injury receiving RPV-free or RPV-based therapy. Black arrows indicate positive non-parenchymal cells. Scale bar = 0.1 mm.

To directly analyze liver tissue, a liver biopsy was performed and the activation of STAT1 was assessed by the presence of its nuclear expression. Interestingly, nuclear STAT1 expression in non-parenchymal cells revealed enhanced activation of this transcription factor in hepatic sections from patients with identified liver injury who were on RPV-based therapy, as demonstrated by representative images of different liver biopsies (Figure 1) and quantification of the STAT1 signal (Table 1). The protective effect of this treatment, in which it promotes STAT1-dependent HSC inactivation, was observed in patients at different stages of MASLD, from mild/intense steatosis to steatohepatitis or fibrosis. Interestingly, the increase in STAT1 activation induced by RPV-based therapy was also evident in F0 patients, probably due to the presence of steatosis or steatohepatitis among these individuals.

## 4. Discussion

Currently, the priority when managing PLWH is to improve their quality of life by optimizing cART, especially regarding metabolic and hepatic disorders. As liver fibrosis and cirrhosis are more frequent in HIV-infected adults than in the general population [12,13], identification of safe or beneficial cART regimens is vital to avoid the development of advanced chronic liver disease in these patients. This study provides evidence that RPV-induced activation of STAT1 in non-parenchymal cells attenuates liver injury in PLWH and MASLD, a result that is in line with our previous data suggesting a hepatoprotective role of RPV [3]. Increasing evidence suggests that STAT1 could be a new therapeutic target for treating liver fibrosis. The activation of this transcription factor has previously been associated with cell death and the reduction of cell proliferation in different liver cell populations [14]. Specifically, enhanced STAT1 activation in hepatic non-parenchymal cells, particularly in HSCs, has been related to the inhibition of HSC proliferation, reduction in TGF-β signaling, and stimulation of NK cell killing of activated HSCs, all of which attenuate liver fibrosis [4]. Our previously reported results demonstrate that RPV promotes beneficial effects in different murine models of liver injury, including a nutritional model of MASLD; importantly, STAT1-mediated pathways have been shown to be fundamental for eliminating activated HSCs via apoptosis and for decreasing collagen production [3]. NNRTI-containing therapies have recently been associated with a higher decrease in liver stiffness than other cART combinations in HIV/HCV-coinfected patients receiving DAA-based therapy. Although authors did not separately analyze the different NNRTIs administered to this population, almost 72% of the patients receiving these combinations were actually receiving RPV, so we cannot rule out that a significant part of the beneficial effects observed were due to this drug [15]. In line with these results, RPV-based regimens have been reported to significantly reduce liver stiffness, as measured by TE, in another cohort of HIV/HCV-coinfected patients cured of chronic hepatitis C [16]. As these two studies were carried out in HCV/HIV-coinfected subjects that received DAA-based therapy, their results cannot be directly compared to our data. However, we did observe a similar trend of lower TE measurements in patients receiving RPV-based regimens, and of non-significant alterations indicative of improved liver function and lipid profile. Altogether, these data suggest a role for RPV in liver injury resolution and regeneration, in addition to its antiretroviral effect. In addition, preliminary data from a large prospective cohort (CoRIS) of PLWH have shown that a TDF + (3TC/FTC) + RPV regimen has an excellent metabolic profile and could have a protective effect on insulin resistance, type 2 diabetes mellitus, and hepatic steatosis, as measured by non-invasive markers [17,18].

Despite this being a non-randomized, non-paired study, the general characteristics of our patients were similar in both groups (age, sex, race). Of note, all patients were male; this was not due to a deliberate exclusion of female subjects, but rather because of the normal composition of the current cohorts of PLWH in our country, of which more than 85% are men [19]. This constitutes a limitation of this study, as we cannot assume that these same results would be obtained in females. Another important limitation is the low number of patients recruited in each of the specific groups. This may have been a hindrance to us obtaining statistically significant results in parameters reported to be predictors of end-stage liver disease risk in previous studies, such as triglycerides, liver enzymes, or TE levels; however, it is important to note that all these parameters were reduced in RPV-treated patients versus subjects not exposed to this NNRTI. As PLWH present heterogenous characteristics, especially regarding past and current cART regimens and their metabolic and hepatic background [20,21], prospective studies with larger, well-controlled cohorts should be performed to corroborate our results and confirm the long-term impact of RPV-based regimens to reduce liver injury and MASLD progression.

To conclude, HIV-infected patients with MASLD on RPV-based therapy display increased STAT1 activation in non-parenchymal cells, suggesting there is ongoing HSC inactivation and apoptosis, which reduces the progression of hepatic damage. Our results suggest that RPV-based cART is especially indicated in HIV-infected patients at risk of MASLD or MASLD-derived liver injury if liver inflammation and fibrosis are to be prevented. Although further studies are necessary to expand on these preliminary findings, the present results are of great clinical relevance due to the lack of specific treatments for MASLD, and because they highlight the potential of RPV as a new therapeutic strategy in the field of liver diseases.

## Figures and Tables

**Table 1:** Subject characteristics and adjusted analysis of LnSTAT1 expression.

				ALL (N = 42)	RPV Non-Exposed (N = 23)	RPV Exposed (N = 19)	*p*
Age (years) *				49 (44; 54)	51.6 (40; 55)	47.8 (45; 51)	0.44
Male gender, N (%)				42 (100)	23 (100)	19 (100)	---
Caucasian, N (%)				34 (89.5)	21 (95.5)	13 (81.3)	0.22
HIV viral load < 50 cop/mL, N (%)				42 (100)	23 (100)	19 (100)	---
HIV infection time (years) *				12.2 (7; 21)	15.4 (7; 23)	9.4 (6; 18)	0.13
CD4 cell count (cell/µL) *				802 (608; 940)	816 (651; 1015)	774 (558.6; 892)	0.44
Nadir CD4 cell count (cell/µL) * Time on current cART (months) *				265 (188; 438) 14 (8; 34)	262 (188; 413) 10 (6; 18)	318 (187; 441) 30 (12; 38)	0.64 **0.02**
Body Mass Index (kg/m^2^) *				28.8 (25.3; 30.7)	28.7 (25.2; 30.7)	28.9 (25.3; 30.8)	0.79
AST (UI/L) *				42 (35; 58)	44 (35; 56)	39 (33; 61)	0.67
ALT (UI/L) *				76 (52; 87)	81 (52; 89)	66 (50; 84)	0.44
GGT (UI/L) *				56.5 (33; 126)	56 (33; 126)	57 (33; 154)	0.77
Glucose (mg/dL) *				104 (96; 110)	102 (99; 108)	105 (89; 112)	0.75
Cholesterol (mg/dL) *				171.5 (156; 203)	167 (152; 214)	175 (162; 195)	0.66
LDL-C (mg/dL) *				107 (87; 124)	96 (86; 131)	114 (93; 124)	0.44
HDL-C (mg/dL) *				37 (31; 42)	37 (31; 42)	37 (30; 44)	0.82
Triglycerides (mg/dL) *				152.5 (110; 225)	155 (135; 231)	125 (79; 225)	0.18
Transient elastography (TE, kPa) *				6.4 (4.4; 8.9)	7.6 (4.5; 10.1)	5.6 (4.1; 6.6)	0.11
TE > 7 kPa, N (%) CAP (dB/m) *				18 (43.9) 302 (257; 347)	14 (60.9) 306 (261; 353)	4 (22.2) 290 (243; 326)	**0.025** 0.33
Diabetes mellitus or IFG ^?^, N (%)				29 (69)	17 (73.9)	12 (63.2)	0.52
Arterial hypertension, N (%)				25 (59.5)	15 (65.2)	10 (52.6)	0.53
Hypercholesterolemia, N (%)				19 (45.2)	13 (56.5)	6 (31.6)	0.13
Hypertriglyceridemia, N (%)				23 (54.8)	14 (60.9)	9 (47.4)	0.53
Mixed dyslipidaemia, N (%)				19 (63.3)	13 (65)	6 (60)	1.00
Cardiovascular events, N (%) ^$^				1 (2.4)	1 (4.3)	0 (0)	1.00
Metabolic syndrome, N (%) ^&^				25 (59.5)	15 (65.2)	10 (52.6)	0.53
Lipid-lowering drugs, N (%)				16 (38.1)	12 (52.2)	4 (21.1)	0.06
Glucose-lowering drugs		Metformin		10 (23.8)	5 (21.7)	5 (26.3)	1.00
		Insulin		2 (4.8)	2 (8.7)	0 (0)	0.49
**Histological findings**							
No steatosis, N (%)				5 (11.9)	1 (4.3)	4 (21.1)	
Steatosis, N (%)		Yes (any degree)		37 (88.1)	22 (95.7)	15 (78.9)	0.16
		Mild (<33%)		19 (45.2)	12 (52.2)	7 (36.8)	
		Moderate (33–66%)		11 (26.2)	5 (21.7)	6 (31.6)	
		Severe (>66%)		7 (16.7)	5 (21.7)	2 (10.5)	0.25
Steatohepatitis (steatosis only), N (%)				25 (67.6)	15 (68.2)	10 (66.7)	1.00
Fibrosis, N (%)		No		24 (57.1)	14 (60.9)	10 (52.6)	
		F1		14 (33.4)	7 (30.4)	7 (36.9)	
		F2		1 (2.4)	1 (4.3)	0 (0)	
		F ≥ 3		3 (7.1)	1 (4.3)	2 (10.5)	0.80
			**ALL**	**RPV non-exposed**	**RPV exposed**	**Mean** **Difference**	***p***
**LnSTAT-1**	All §		9.8 (9.4; 10.3)	9.1 (8.3; 9.9)	10.6 (9.7; 11.4)	1.47 (0.05; 2.9)	**0.04**
	Steatohepatitis	No §	9.9 (9.2; 10.6)	9.7 (8.5; 10.8)	10.2 (9.1; 11.3)	0.52 (−1.24; 2.27)	0.55
		Yes §	9.9 (9.3; 10.4)	8.8 (8.0; 9.7)	10.9 (9.8; 12.0)	2.07 (0.46; 3.67)	**0.01**
	Steatosis > 30%	No §	9.8 (9.2; 10.3)	9.2 (8.2; 10.2)	10.3 (9.3; 11.3)	1.12 (−0.50; 2.74)	0.17
		Yes §	9.9 (9.2; 10.6)	8.9 (7.9; 9.9)	10.9 (9.7; 12.2)	2.06 (0.25; 3.87)	**0.03**
	Fibrosis > F0	No ¥	9.8 (9.3; 10.4)	9.0 (8.2; 9.9)	10.7 (9.7; 11.7)	1.66 (0.21; 3.11)	**0.03**
		Yes ¥	9.8 (9.1; 10.4)	8.9 (7.8; 9.9)	10.6 (9.5; 11.7)	1.77 (0.02; 3.53)	**0.04**

* Median (percentile ^25th^; percentile ^75th^); ^?^ Impaired Fasting Glucose; ^$^ Cardiovascular events: ischemic heart disease and ischemic stroke; ^&^ Metabolic syndrome: diagnosis criteria by Adult Panel Treatment (ATP) III definition: 3 of the 5 risk abnormalities (waist circumference ≥ 102 cm in men or ≥88 cm in women; fasting glucose ≥ 100 mg/dL or on treatment; triglycerides ≥ 150 mg/dL; HDL < 40 mg/dL in men and <50 mg/dL in women, or on treatment; and systolic blood pressure ≥ 130 mmHg and/or diastolic blood pressure ≥ 85 mmHg; or on treatment). § Marginal mean (95% CI) adjusted for exposure time to RPV (months) and BMI (General Linear Model). ¥ Marginal mean (95% CI) adjusted for exposure time to RPV and EFV (months) (General Linear Model). *p*: *p*-value obtained in the contrast test (Chi-squared, Fisher’s exact test, Mann–Whitney, Student’s *t* test or GLM, as appropriate).

## Data Availability

The raw data supporting the conclusions of this article will be made available by the authors on request.
